# Assessment of the capacity to consent to treatment in patients admitted to acute medical wards

**DOI:** 10.1186/1472-6939-10-15

**Published:** 2009-09-02

**Authors:** Sylfa Fassassi, Yanik Bianchi, Friedrich Stiefel, Gérard Waeber

**Affiliations:** 1 Service of Liaison Psychiatry, CHUV-University hospital, Lausanne, Switzerland; 2 Department of Internal Medicine, CHUV-University hospital, Lausanne, Switzerland

## Abstract

**Background:**

Assessment of capacity to consent to treatment is an important legal and ethical issue in daily medical practice. In this study we carefully evaluated the capacity to consent to treatment in patients admitted to an acute medical ward using an assessment by members of the medical team, the specific Silberfeld's score, the MMSE and an assessment by a senior psychiatrist.

**Methods:**

Over a 3 month period, 195 consecutive patients of an internal medicine ward in a university hospital were included and their capacity to consent was evaluated within 72 hours of admission.

**Results:**

Among the 195 patients, 38 were incapable of consenting to treatment (unconscious patients or severe cognitive impairment) and 14 were considered as incapable of consenting by the psychiatrist (prevalence of incapacity to consent of 26.7%). Agreement between the psychiatrist's evaluation and the Silberfeld questionnaire was poor (sensitivity 35.7%, specificity 91.6%). Experienced clinicians showed a higher agreement (sensitivity 57.1%, specificity 96.5%). A decision shared by residents, chief residents and nurses was the best predictor for agreement with the psychiatric assessment (sensitivity 78.6%, specificity 94.3%).

**Conclusion:**

Prevalence of incapacity to consent to treatment in patients admitted to an acute internal medicine ward is high. While the standardized Silberfeld questionnaire and the MMSE are not appropriate for the evaluation of the capacity to consent in this setting, an assessment by the multidisciplinary medical team concurs with the evaluation by a senior psychiatrist.

## Background

Assessment of the capacity to consent to treatment is an important legal and ethical issue in medicine. Providing treatment against the wishes of a patient capable of consenting to treatment violates the principle of patient autonomy and can often violate physician beneficence [1]. Accurate assessment of the patient's capacity to consent is therefore most important for decisions regarding medical treatments which may have severe side effects or even result in fatal outcomes [2]. The capacity to consent to treatment requires the ability to understand and retain information, to use this information as part of the decision-making process and to make free choices. This capacity is specific to a particular decision and can be unstable. In busy clinical practice, however, capacity to consent is often presumed unless the patient refuses treatment [3] or shows obvious signs of cognitive failure or mental disorder. This policy may be the best acceptable clinical and ethical approach considering that it may be very difficult to assess the capacity to consent in a situation where patients have not yet been exposed to a specific choice related to their health.

While capacity to consent to treatment depends on the above-mentioned patient factors, the ability to realize this capacity also depends on physician factors [4], such as skills in explaining relevant medical facts adapted to the patient's educational and cultural background. Several studies have demonstrated difficulties associated with the assessment of patients' capacity to consent to treatment [5,6] by clinicians who tend to rely on informal clinical impressions [7]. The aim of the present study was (i) to identify the prevalence of patients lacking of capacity to consent to treatment within the first 72 hours of admission to a general internal medicine ward of a university hospital and (ii) to compare a standardized assessment by means of the Silberfeld questionnaire with the assessment by a multidisciplinary medical team or by a senior psychiatrist. Previous studies evaluating capacity to consent were limited to homogeneous samples based on age [8], pathology (psychiatric disorders [9-11], neurologic disorders [12]), or medical setting (patients included in research protocols or treated in the ambulatory care setting^n^[13]).

## Methods

The study was conducted in a general internal medical ward at Lausanne University Hospital (CHUV) during a three-month period from June 1 to August 31, 2007. Assessment of the capacity to consent to treatment was conducted during the first 72 hours following admission. The research protocol was accepted by the hospital's ethics committee and written consent was obtained from all participating patients (and/or from their relatives and/or general practitioner if capacity to consent was profoundly altered).

### Participants

Patients who refused to participate; who could not read or speak French (and therefore were unable to fill in the Silberfeld questionnaire) or who had major haemodynamic instability were excluded. All other admitted patients were included in the study. Patients with an obvious lack of capacity to consent, such as unconscious patients or patients who exhibited severe cognitive impairment, (i.e. unable to communicate, to recall their date of birth or their name) were considered without formal evaluation as "incapable to consent to treatment" (as suggested by the hospital's ethics committee, informed consent of these patients was obtained from their general practitioner or their relative).

### Assessment

Capacity to consent is specific to a decision and can vary over time; a patient is therefore competent or not with respect to a specific decision and for a given moment in time. The capacity to consent to the treatment or investigation proposed during hospitalisation was evaluated specifically for each patient according to the clinical situation. Each patient was assessed by a research fellow by means of the Mini-Mental State Examination (MMSE) and the Silberfeld questionnaire. The French version of the MMSE was developed by the Working Group on Cognitive Evaluations [14]; the original version was developed by Folstein *et al (1975) *as a method for grading cognitive impairment (score between 0-30, with 0 indicating the most severe cognitive disturbances and a score above 23/30 indicating no severe cognitive impairment) [15]. The MMSE is a brief, easily administered test of several cognitive functions which can play a significant role in the process of decision-making and, therefore, to consent. The test's validity and reliability have been demonstrated in psychiatric, neurological, geriatric, and other medical settings [16].

The Silberfeld questionnaire assesses adult patient's capacity to consent to clinical treatment using two vignettes describing common clinical situations [17]. Each vignette is read to a patient followed by nine questions concerning the vignette which leads to a score between 0 and 10 points (lower scores indicating an impaired capacity to consent). The same nine questions are then applied to the actual medical situation of the patient. Filling in the Silberfeld questionnaire takes about 30 to 45 minutes. The authors suggest that patients with a score equal or superior to 6 are capable of consenting to treatment.

The medical team, consisting of the resident or fellow, his supervisor (a chief resident or a senior physician), the nurse in charge of the patient and the referring general practitioner were asked to indicate if the patient had or had not (yes/no) the capacity to consent to investigations or treatment.

The psychiatric assessment by a senior psychiatrist was based on the guidelines described by Applebaum and Grisso [18], which evaluate the patient's ability 1) to appreciate the situation and its consequences, 2) to understand the relevant information, 3) to manipulate the information rationally and 4) to communicate and maintain a choice. The psychiatrist also looked for evidence of psychopathology affecting capacity, such as delusions. The first psychiatric evaluations were made in the presence of a psychiatrist who co-developed local guidelines for the assessment of patient decision making capacity in the general hospital [19].

The research fellow who applied the Silberfeld and who asked the medical team and the general practitioner about their clinical impression and the psychiatrist who evaluated the patients worked independently and did not have access to the results of the different assessments.

Sociodemographic and medical information were obtained from the medical charts of the participating patients.

### Statistical analysis

Correlation between Silberfeld and MMSE scores and evaluations of the capacity to consent by members of the medical team, referring general practitioner and the psychiatrist were analysed using the SPSS version 9.0. To identify overall agreement with the psychiatric assessment, the receiver-operating curve (ROC) was calculated. Data were analysed following the cut-off scores of the MMSE and Silberfeld scores as recommended in the literature [17,20].

## Results

### Sample

**Figure 1:**
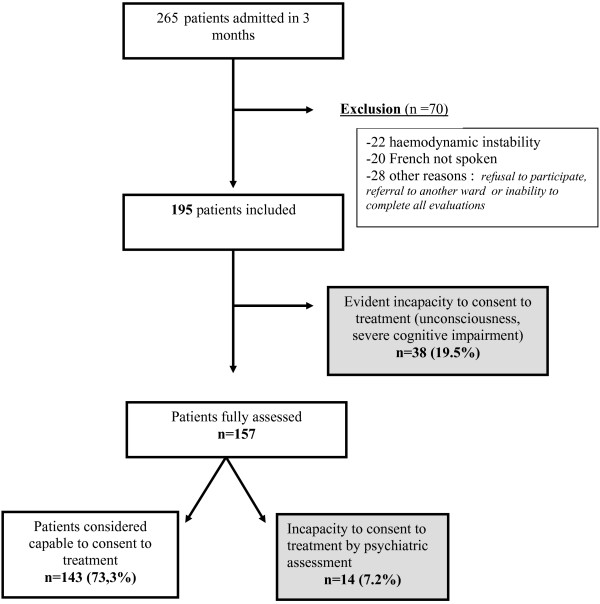
**The figure describes the recruitment flowchart with the number of patients potentially eligible, the number of patients excluded and the main reason for exclusion**. For the included patients the number (and the percentage) of patients considered to have (i) an evident incapacity to consent to treatment, (ii) an incapacity to consent to treatment based on the psychiatric assessment (iii) and a capacity to consent to treatment are listed.

The sample is described in figure 1.

### Prevalence of incapacity to consent for treatment

Of the participating patients (n = 195), 26.7% (n = 52) were considered as incapable to consent: 38 were unconscious, unable to communicate or cognitively impaired to a degree that they were unable to recall their name or date of birth;14 qualified as incapable according to the psychiatric assessment.

### Study sample

Sociodemographic and medical characteristics of evaluated patients (n = 157) are listed in Table 1; their mean age was 68.6 years old (SD 18.2), 59.2% (n = 93) were women, 42.7% (N = 67) were married or lived with someone, 14% (n = 22) were single, 15.3% (n = 24) divorced and 28% (n = 44) widowed. The principal reasons for hospitalisation were pulmonary 29.3% (n = 46), cardiovascular 24.8% (n = 39) and digestive 21% (n = 33) disorders. No association between sociodemographic variables and capacity to consent was identified.

**Table 1:** Personal and clinical characteristics of patients (n = 157) admitted to the general internal medicine wards who were assessed for mental capacity to consent to treatment.

Age in years +/- Standard deviation	68.6	+/- 18.2
		

	%	N

Male gender	40.8	64

		

Highest level of education		

Primary School	80.9	127

High school	10.2	16

College/University	8.9	14

		

Place of residence before admission		

Independent home or flat	73.9	116

Health care at home	24.2	38

Nursing home	1.9	3

		

Marital Status		

Married/in couple	42.7	67

single	14.0	22

Divorced	15.3	24

Widowed	28.0	44

		

Reason for hospitalization		

cardiovascular disorder	24.8	39

pulmonary disorder	29.3	46

digestive disorder	21.0	33

Others disorders (renal, urogenital, metabolic, osteoarticular, neurologic, etc.)	24.9	39

		

Co-morbidities		

cardiovascular	70.0	110

pulmonary	38.9	61

digestive	39.5	62

renal	38.2	60

metabolic	38.9	61

osteoarticular	30.6	48

urogenital	23.6	37

neurologic	19.1	30

psychiatric	15.9	25

### MMSE

With the MMSE cut-off score of 23, the prevalence of patients with cognitive impairment was 15.3% (n = 24/157). A MMSE score below 23 increased the probability of incapacity based on the psychiatric assessment. However 16 patients were capable of consenting despite cognitive impairment on the MMSE.

### Silberfeld questionnaire

**Figure 2:**
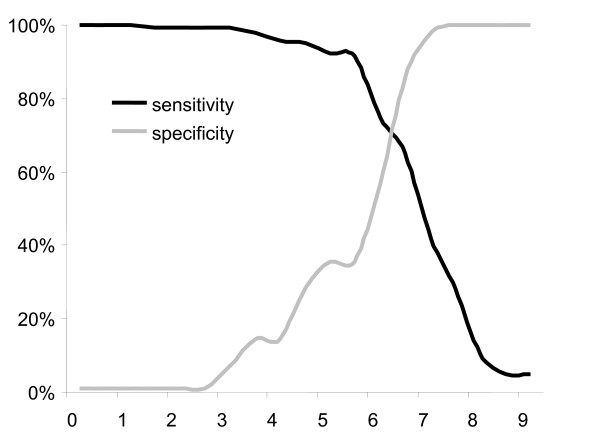
**The sensitivity and specificity of the Silberfeld score is compared to the psychiatric assessment for the evaluated patients (n = 157)**. As shown in the figure, a cut off score equal or superior to 6 has the best sensitivity and specificity.

Agreement between the psychiatric assessment and the Silberfeld questionnaire was poor (kappa 0.249), with a sensitivity of 35.7% and a specificity of 91.6% (see Figure 2); using the Silberfeld score, 12 patients were classified false positive and 9 false negative.

### Opinions of the medical team

We considered the opinions of the medical team (resident, nurse and supervisor) individually and then the majority decision of the medical team. The majority decision showed the highest agreement with the psychiatric assessment (specificity and sensitivity of 94.3% and 78.6%, respectively); when compared to the psychiatric assessment the opinion of the general practitioner showed a specificity and sensitivity of 97.4% and 36.4%, respectively. The sensitivity and specificity of the various evaluations of capacity to consent to treatment are shown in Table 2.

**Table 2:** Sensitivity and specificity of the Silberfeld, the MMSE and various clinical opinions with regards to patients' (n = 157) capacity to make decisions compared to the psychiatrist assessment.

	Sensitivity (%)	Specificity (%)
Silberfeld	35.7	91.6

MMSE	57.1	88.8

Resident	35.7	96.5

Nurse	50.0	94.4

Chief resident internist (CRI)	57.1	96.5

Referring general practitioner	36.3	97.8

CRI, Resident and Nurse (majority decision)	78.6	94.3

Disagreement among the medical team was observed for 22 patients (14% of the sample), with half of them (n = 11) considered as incapable of consenting to treatment by the psychiatrist.

## Discussion

The aim of this study was to identify the prevalence of patients lacking capacity to consent to treatment within the first 72 hours of admission to a general internal medicine ward of a university hospital. Of the patients (n = 52; 26.7%) who were identified to lack capacity to consent, a majority (n = 38; 19.5%) exhibited obvious incapacity to consent, (unconsciousness, severe cognitive impairment); an additional 14 (7.2%) patients lacking capacity to consent (almost a third of all patients lacking capacity to consent) were identified by the psychiatrist. These findings illustrate that besides the easily identifiable patients, some patients have to be evaluated in order to determine their incapacity to consent.

70 patients had to be excluded due to the impossibility of evaluating them (haemodynamic instability, French not spoken) or their refusal to participate to a study. This is a limitation of the study since prevalence of patients unable to consent may have been influenced by the excluded patients; however it is not very probable that all of them, especially the haemodynamically unstable, were competent. Patients who refused did so for various reasons (fatigue, "no time", etc.) Their refusal was not explicitly clarified and documented, since for ethical reasons their refusal had to be respected. Although we did not know how many of them refused due to incompetence, we do not believe this was often the case.

The second question of our study was to compare a standardized assessment (the Silberfeld questionnaire, a tool suggested by our institutional directive) with the assessment by a multidisciplinary medical team or by a senior psychiatrist. Our main finding was that the clinical team is more accurate in assessing capacity to consent than either an individual or a standardized test.

Confirming a previous study, no association was found between demographic variables (age, education etc.) and capacity to consent to treatment [21]. While capacity to consent may possibly be the same across gender and educational variables, in elderly patients, cognitive deficits may be more prevalent and thus influence the capacity to consent. Our sample may not be large enough to detect such differences.

Several studies have reported the inaccuracy of the standardized Mini Mental State Exam in determining capacity to consent to treatment [7,22,23]. In line with these results, we observed that the MMSE showed a specificity of 57.1% and a sensitivity of 88.8%. For example one patient with an MMSE score of 29 was found to lack capacity to consent by the psychiatrist; this patient refused the investigation of a breast tumour because of delusional beliefs.

If a recommended Silberfeld score of 0-6 had been utilized to identify incapacity to consent, 28% of the 195 included patients would have been classified as lacking capacity to consent (a similar percentage to the prevalence found by the psychiatrist); however, the specificity of the Silberfeld score was poor. A previous study revealed that clinical impressions (treating physician) were inaccurate in determining capacity to consent to treatment [7]. For this reason, we included a decision-support tool for the assessment of the capacity to consent in this study. We are aware that the Silberfeld does not represent the most accurate instrument to evaluate competence We chose the Silberfeld questionnaire because the institutional directive of the hospital suggests it be used in daily clinical work (other questionnaires supporting clinical judgement were considered too time consuming. Although the Silberfeld questionnaire offers guiding principles to assess capacity to consent [24], our findings suggest that this tool is not appropriate for the acute care setting. Since the aim of the study was not to compare different questionnaires for the assessment of capacity to consent, the question whether other specific questionnaires designed to assist the clinician would have produced better results remains unanswered.

The opinions of clinicians, especially senior physicians, were more accurate than the Silberfeld questionnaire. This result confirms a prior study reporting that experienced physicians were more likely to make accurate assessments of capacity to consent than younger physicians, but that their assessments could still be improved [25]. In reviewing the cases with disagreement between supervising physicians and the psychiatric assessment, we found that fluctuation of patients' clinical status could partially explain the differences: of the 11 participants who were falsely classified as capable of consenting by the supervisors, two showed a fluctuating clinical status (delirium, acute confusional state). A decision shared by the different clinical team members was the best predictor for agreement with the psychiatrist. However, if disagreement among the clinical team occurs (i.e. disagreement between one of the residents, chief residents or nurses) a psychiatric consultation may be useful, since half of these patients (11/22) were found to be incapable of consenting.

The fact that the general practitioner may not have seen the patient for some time may explain the doctor's relatively poor performance in assessing the patient's capacity to consent. However, it is important to raise the consciousness of general practitioners with regard to this issue, since many of them were surprised and puzzled when asked about their patient's capacity to consent to treatment

The psychiatrist assessing patients' capacity to consent was a senior staff member (SF). Another limitation of this study may arise from the fact that all the results were compared to a single psychiatrist's opinion. However, the study psychiatrist has been specifically trained and based her clinical judgment on a standardised guideline. She was also supervised for the first evaluation by an experienced liaison psychiatrist (FS) who participated in the development of the local guidelines for the assessment of capacity to consent.

Our study was limited to the French speaking patients; indeed 7.5% of 265 patients were excluded due to language barriers. The fact that the medical setting was not able to provide sufficient support for communication (translation, cultural mediation), could increase the prevalence of patients lacking of ability to realise their capacity to consent to treatment.

## Conclusion

Given the high percentage of patients incapable of consenting to treatment and the observed difficulty of the medical staff in determining the capacity to consent, there is a clear need for reliable and valid assessment methods of patients admitted to an acute medical ward. This study demonstrated that a specific tool, such as the Silberfeld questionnaire, is less useful than an interdisciplinary evaluation by clinicians. Other instruments, such as the MacArthur Competence Assessment Tool-Treatment (MacCAT-T) [26] will have to be evaluated and compared to other methods for the assessment of capacity to consent in future studies. The medico-legal contexts with regard to capacity to consent may vary in different countries but the capacity to consent remains an important ethical and legal aspect of patient care in all settings. Our study demonstrates that standardized tools, which can evaluate patients' capacity to consent, and which have been proven to be effective in identifying patients unable to consent patients, are currently lacking. Since a clinical judgement based on a shared interdisciplinary evaluation appears to be the best available option to respect ethico-legal obligations to assess patient capacity, a sound understanding of consent and its accurate evaluation in practice should form part of pre and postgraduate training.

## Competing interests

The authors declare that they have no competing interests. They declare themselves to be independent of funders.

## Authors' contributions

SF performed the psychiatrist assessment, participated in the statistical analysis and drafted the manuscript. YB carried out the assessment by the Mini-Mental State Examination (MMSE) and the Silberfeld questionnaire, recorded the medical team and the general practitioner's clinical impression, and participated in the statistical analysis. FS and GW conceived the study and participated in its design and coordination. All authors read and approved the final manuscript.

## Pre-publication history

The pre-publication history for this paper can be accessed here:


